# Isoform-Specific Properties of Orai Homologues in Activation, Downstream Signaling, Physiology and Pathophysiology

**DOI:** 10.3390/ijms22158020

**Published:** 2021-07-27

**Authors:** Adéla Tiffner, Isabella Derler

**Affiliations:** Institute of Biophysics, JKU Life Science Center, Johannes Kepler University Linz, A-4020 Linz, Austria; adela.tiffner@jku.at

**Keywords:** STIM, Orai, Orai isoforms, structure-function relationship, therapeutic approaches

## Abstract

Ca^2+^ ion channels are critical in a variety of physiological events, including cell growth, differentiation, gene transcription and apoptosis. One such essential entry pathway for calcium into the cell is the Ca^2+^ release-activated Ca^2+^ (CRAC) channel. It consists of the Ca^2+^ sensing protein, stromal interaction molecule 1 (STIM1) located in the endoplasmic reticulum (ER) and a Ca^2+^ ion channel Orai in the plasma membrane. The Orai channel family includes three homologues Orai1, Orai2 and Orai3. While Orai1 is the “classical” Ca^2+^ ion channel within the CRAC channel complex and plays a universal role in the human body, there is increasing evidence that Orai2 and Orai3 are important in specific physiological and pathophysiological processes. This makes them an attractive target in drug discovery, but requires a detailed understanding of the three Orai channels and, in particular, their differences. Orai channel activation is initiated via Ca^2+^ store depletion, which is sensed by STIM1 proteins, and induces their conformational change and oligomerization. Upon STIM1 coupling, Orai channels activate to allow Ca^2+^ permeation into the cell. While this activation mechanism is comparable among the isoforms, they differ by a number of functional and structural properties due to non-conserved regions in their sequences. In this review, we summarize the knowledge as well as open questions in our current understanding of the three isoforms in terms of their structure/function relationship, downstream signaling and physiology as well as pathophysiology.

## 1. Introduction

Ca^2+^ ions are versatile cytosolic messengers that control a myriad of coordinated cellular processes. In the quiescent state, the intracellular Ca^2+^ concentration is very low, in the range of 0.1 µM, whereas on the extracellular side and in intracellular organelles, such as the endoplasmic reticulum (ER) and the mitochondria, the Ca^2+^ levels are high, in the range of 1–2 mM. Activation of the cell, e.g., by receptor-ligand binding, triggers the activation of Ca^2+^ selective ion channels and cytosolic Ca^2+^ levels enhance as a consequence. Depending on the stimulus, Ca^2+^ signals develop in various patterns, ranging from transient to stable signals. This ensures the versatility of the Ca^2+^ ion in regulating a wide variety of biological processes, from the short term, such as secretion, to the long term, such as gene transcription or proliferation. This diversity of Ca^2+^ signaling mechanisms is further established by a toolbox of Ca^2+^ sensing, Ca^2+^ buffering, Ca^2+^ binding and Ca^2+^ transporting proteins, which can act in a cell type-specific manner [1,2,3,4].

A prominent Ca^2+^ entry pathway into the cell is represented by the Ca^2+^ release-activated Ca^2+^ (CRAC) ion channel [5,6,7,8,9,10,11,12,13,14], which is unique due to its composition and structure. It consists of two transmembrane proteins, the Ca^2+^ sensor STIM1 located in the ER and the Ca^2+^ ion channel Orai1 situated in the plasma membrane. Defective signaling mechanisms of the CRAC channel proteins (STIM1, Orai1) can lead to diseases such as severe combined immunodeficiency, thrombocytopenia, tubular aggregate myopathy, ectodermal dysplasia or cancer [11,15,16]. A variety of CRAC channel blockers is currently available; however, only a few have reached clinical trials and none are used in clinics for medical therapy [17,18]. The main problems of the currently available blockers are their low selectivity, high toxicity or unexpected pharmacology [17,18]. It is worth noting that the STIM family consists of two homologues, STIM1 and STIM2, while the Orai family includes three isoforms, Orai1, Orai2 and Orai3 [19,20]. While Orai1 has been attributed a universal role in various tissues, both in health and disease [21,22,23,24,25], Orai2 and Orai3 have been increasingly identified as significant in human body functions and dysfunctions since their discovery [26,27,28,29,30,31,32,33].

To optimize available or to develop novel, target-specific therapeutic strategies, a detailed understanding of the CRAC channel components and their isoforms is required. In this review, we highlight the current knowledge on isoform-specific properties and physiological as well as pathophysiological roles of the three Orai homologues.

## 2. Overview of Store-Operated Ca^2+^ Entry

In a wide variety of cell types, intracellular Ca^2+^ signals arise from Ca^2+^ influx via store-operated Ca^2+^ ion channels. They are controlled by the amount of Ca^2+^ in the intracellular Ca^2+^ stores, typically the ER. Their activation mechanism is considered to be a biphasic process in which depletion of the ER Ca^2+^ store is associated with Ca^2+^ influx from the extracellular side. The best characterized store-operated Ca^2+^ ion channel is the CRAC channel [11,34,35].

In detail, receptor stimulation at the cell membrane leads to the activation of a signaling cascade that activates phospholipase C via a tyrosine kinase cascade or G proteins to hydrolyze phosphatidylinositol 4,5-bisphosphate (PIP_2_) and generate inositol 1,4,5-trisphosphate (IP_3_). IP_3_ binds to the IP_3_ receptors (IP_3_R) in the ER membrane, which releases Ca^2+^ from the ER lumen to the cytosol and activates the CRAC channel [11,34,35].

The STIM1 proteins located in the ER membrane sense ER luminal Ca^2+^ via their N-terminal EF-hand-sterile-α-motif (SAM) domains [36,37,38,39,40,41]. A reduction in Ca^2+^ levels in the ER triggers the release of Ca^2+^ originally bound to STIM1. Subsequently, STIM1 proteins undergo a conformational change and oligomerize [37,38,42,43]. Store depletion-induced structural changes propagate from its N-terminal strand to its single TM domain and finally to the C-terminus [44,45,46,47,48]. In particular, STIM1 C-terminus adopts a tight conformation in the quiescent state, which fully extends upon activation to bind to Orai channels in the plasma membrane [45,49,50,51]. STIM1 C-terminus contains subsequent to its TM domain three coiled-coil regions (CC1, CC2, CC3) which is followed by a flexible strand [36,52]. An inhibitory clamp, especially of the 1st and 3rd coiled-coil region, contributes to the maintenance of the quiescent state [45,49,50,51]. In the active state, the CC2-CC3 segment, known as the STIM–Orai-activating region (SOAR) or Ca^2+^ release-activated Ca^2+^-activating domain (CAD) region, couples to Orai [53,54], while CC1 and CC3 are supposed to stabilize the extended conformation via interacting and oligomerizing with each other [9,48].

Since the discovery of the three Orai proteins, Orai1 has been the most investigated. It is ubiquitously expressed and triggers together with STIM1 the “classical” store-operated Ca^2+^ influx in many cells, including immune cells, cardiomyocytes, airway, vascular smooth muscle cells, endothelial cells and melanocytes [21,22,23,24,25]. Hence, Orai1 is critical in a multitude of cellular processes, as apparent from a variety of gain-of-function (GoF) and loss-of-function (LoF) mutations within Orai1 that have been linked to diseases such as severe combined immune deficiency [15,21,55]. Interestingly, there is emerging evidence that Orai2 and Orai3 possess critical roles in pathogenesis [26,27,28,29,30,31,32,33]. Especially Orai3 seems to be a major contributor to the development of various types of cancers [26,27,28,29,30,31,32,33] and Orai2 has been identified to fine-tune immune cell response [56].

Overall, it is important to understand the structure and function of the three Orai isoforms. In particular, their distinct structural and functional features, which will be reviewed in the following section, might arise as promising for target-specific therapeutic strategies.

## 3. Similarities and Differences of the Structure of Orai Isoforms

**Figure 1:**
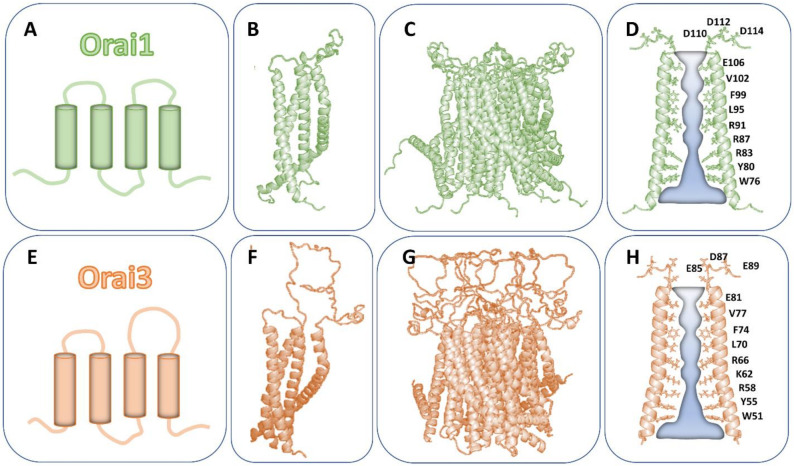
The Orai1 and Orai3 channel assembly. (**A**) The scheme visualizes the 4-TM domain structure of the Orai1 (green) channel with two extracellular and one intracellular loop. Both the N- and C-terminus are located in the cytosol. (**B**) The structure of the Orai1 subunit. (**C**) The side view of the Orai1 channel composed of six subunits from (**B**). (**D**) The pore of the Orai1 channel is highlighted by the most important pore-forming residues. (**E**–**H**) In analogy to Orai1 (**A**–**D**), (**E**) represents the scheme, (**F**) the single subunit, (**G**) the side view and (**H**) the pore of Orai3 (orange).

Structurally, all three Orai proteins consist of four TM domains flanked by the cytosolic N- and C-terminus and linked by two extracellular and one intracellular loop [57,58,59] (Figure 1A–D). Despite a comparable general structure of the Orai subunits, they share only 50–60% overall sequence identity [29]. In detail, only the sequence of Orai TM1 regions is identical, while the other three TM domains are about 81–87% comparable [34] (Table 1).

The cytosolic and extracellular regions show stronger differences in sequence. The N-terminus (aa 1–90) exhibits 34% sequence identity, while the C-terminus (aa 265–301) is about 46% comparable [29] (Table 1). The first extracellular loop (loop1) is 60–80% conserved and differs especially in the sequence of residues with charged side chains [52] (Table 1). The intracellular loop domain (loop2) linking TM2 and TM3 contains 4 (Orai2) and 8 (Orai3) non-conserved residues. This is sufficient to alter its structural properties, as we found by comparing molecular dynamic (MD) simulations for Orai1 and Orai3 [60]. Although the Orai1 loop2 and Orai3 loop2 are composed of identical numbers of amino acids, the flexible portion of this region is longer in Orai1 than in Orai3, resulting in a shorter cytosolic extension of TM2 in Orai1 than in Orai3 [60]. The second extracellular loop region (loop3) connecting TM3 and TM4 is only 20–30% comparable between Orai isoforms [29] (Table 1).

Prior to the first reported Orai structure [61], Orai channels were hypothesized to form tetrameric assemblies [7,8,57,58,59,62,63,64]. In regard to isoform-specific Orai channel assemblies, one study reported, using single-molecule photobleaching, that both Orai1 and Orai3 undergo a dimer-to-tetramer transition upon STIM1-triggered activation. Interestingly, the pharmacological compound 2-aminoethoxydiphenyl borate (2-APB), a well-known activator of Orai3 already independent of STIM1 [65,66,67] (see Section 7), left the dimeric conformation unaffected [7,8,59]. In contrast to the dimeric and tetrameric Orai channel assemblies, the currently available crystal and cryogenic electron microscopy (cryo-EM) structures [61,68,69,70] resolved a hexameric Orai complex (Figure 1E,F). Although all currently available structural resolutions are only from *Drosophila melanogaster* Orai, homology modeling strongly suggests that all human Orai homologues form hexameric complexes [60,71,72]. In line with these structures, concatemeric studies also indicate that functional Orai channels are hexameric complexes [73,74].

Within the hexameric complex, all six TM1 domains are located in the center and form the Ca^2+^ selective ion pore of the Orai channel [61,68,69,70]. At the extracellular side, the ion pore consists of the Ca^2+^ accumulating region (CAR) [72], which is followed by the selectivity filter (E106) and the hydrophobic cavity (L95, F99, V102) [63,75,76]. At the intracellular side of the pore, a basic region extends into the cytosol (Figure 1G,H). Upon pore opening, Ca^2+^ ions are attracted by the CAR region and transferred to the selectivity filter [72]. STIM1 coupling is supposed to induce a rotation of TM1 around the hydrophobic cavity, moving F99 out of and G98 into the pore region [77]. This allows Ca^2+^ permeation through the hydrophobic segment to the basic region. The role of the basic region in Ca^2+^ permeation is still controversial. Structural studies suggest that the residues with positively charged side chains are shielded by negatively charged chloride ions, and thus, either maintaining the closed state [61] or allowing Ca^2+^ permeation [68,78]. The positively charged R91 has been suggested to rotate away from the pore region upon Orai1 activation [71]. Alternatively, it has been reported that the basic residues maintain pore hydration [79]. Key residues in the pore lining TM1 domain of Orai channels are highlighted in Table 2.

The pore region is surrounded by a ring of TM2 and TM3 and at the periphery by TM4 regions connected to the helical C-termini [61] (Figure 1E,F). There is profound evidence that pore opening is allosterically affected by checkpoint residues in TM2, TM3 and TM4 [12,80,81]. All Orai TM domains include residues, which when mutated can lead to GoF independent of STIM1, clearly indicating that they induce pore opening. In line with this, the recent cryo-EM structure [68] of an Orai open state revealed alterations in the pore region, including a rotation of the hydrophobic cavity and dilation of the basic region. Moreover, LoF mutations, especially at the cytosolic helical extension of the TM regions, including most prominently the cytosolic triangles (see Section 4), interfere with pore opening due to reduced hydration of the pore region [12,81]. At the outmost side of the channel complex, the C-terminus is supposed to be connected to TM4 by a bent region [61] (Figure 1C–F), the so-called nexus [82,83], while in the open state, the TM4-C-terminus region is assumed to undergo a conformational change [68,69,70]. To which extent this structural alteration occurs physiologically upon STIM1 coupling is still a matter of debate.

Aside from homomeric Orai assemblies, Orai homologues have been reported to be heteromerized [84], which is in particular crucial in native systems [56,85] (see Section 9). In addition to the store-dependent hexameric Orai channel complex, a pentameric assembly composed of Orai1 and Orai3 subunits has been reported to represent the store-independent, arachidonate regulated Ca^2+^ (ARC) channel [86]. It contains three Orai1 and two Orai3 subunits [58,87,88,89]. Isoform-specific differences of the Orai N-termini (see Section 4) are critical for the switching between a store-operated to a store-independent, exclusively arachidonate activatable ion channel [90]. Furthermore, Leukotriene C_4_ (LTC_4_)-regulated Ca^2+^ (LRC) channels have been reported to form Orai1/3 heteromultimers, altough the ratio of Orai1:Orai3 subunits is currently elusive [91].

Overall, the Orai subunits can form homo- or heteromeric, hexameric complexes to form store-operated Ca^2+^ ion channels, whereas the store-independent ARC channel is a pentameric assembly of Orai1 and Orai3 subunits. A number of isoform-specific differences in the sequence of human Orai isoforms exist. Among those, several ones have already been reported to lead to structural and/or functional differences, which will be described in detail below.

## 4. Isoform-Specific Differences in Orai Channel Function

All three Orai isoforms have in common that they can be activated by STIM1 upon store-depletion. As a result, strongly inwardly rectifying Ca^2+^ selective currents develop, all with a reversal potential of +50 mV [67]. However, they differ in the maximum current magnitude as well as several other biophysical characteristics, which develop due to isoform-specific structural differences within the Orai proteins [92].

**Figure 2:**
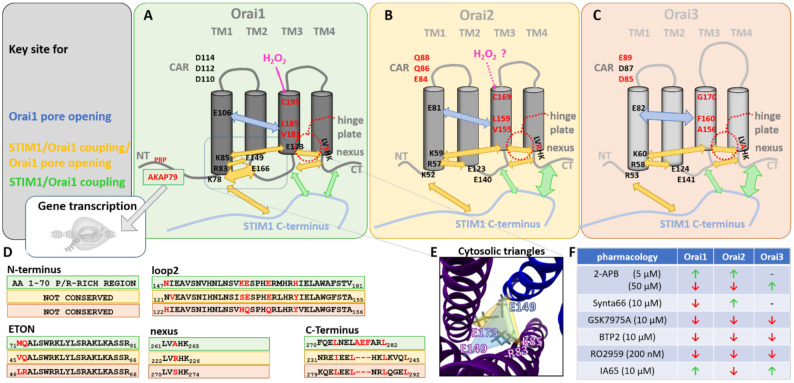
Isoform-specific similarities and differences of Orai homologues. (**A**–**C**) Orai1, Orai2 and Orai3 are shown in comparison with the analogous residues highlighted in black that represent critical conserved amino acids, while the red marked residues are non-conserved positions among the three isoforms. Blue arrows illustrate the regions important for pore opening, yellow arrows are areas important for STIM1/Orai1 coupling and pore opening while green arrows highlight the regions merely responsible for STIM1/Orai1 interaction. Structurally, Orai homologues are rather similar, consisting of four TM domains with N and C termini exposed into the cytosol. It is worth noting that Orai3 possesses a longer loop3 region. (**D**) The sequence alignments display positions of non-conserved residues (in red) within important Orai segments determining distinct isoform-specific behavior. (**E**) The cytosolic triangles containing functional relevant salt-bridge interactions of R83-E149 and K85-E173 within one subunit and K85-E149 between two subunits. (**F**) The table summarizes the activity of three Orai isoforms upon application of different modulators. The small and large green arrows define the slight or robust potentiation, respectively, while red arrows are a slight or robust blockage (n.d. = not determined).

The activation of Orai channels involves in the initial step the direct interaction with STIM1. The indispensable prerequisites for their coupling are their C-termini [43,93]. Moreover, the N-terminal strand and the loop2 region, linking TM2 and TM3, are essential for STIM1-mediated activation [60,94,95,96]. At the level of Orai1, we reported that a region in loop2 contributes via direct coupling to a segment in STIM1 (α3) to transmit the gating signal to the pore region [60]. A defect at the binding interface of STIM1α3 and Orai1 loop2 maintains STIM1/Orai1 coupling but abolishes Orai1 activation [97]. An intact N-terminus, in particular the last 20 amino acids (aa 70–90), is critical to maintain CRAC channel activation and biophysical hallmarks [92,95]. While this is the general STIM1/Orai coupling mechanism for Orai isoforms, the cytosolic regions exhibit isoform-specific differences that are reflected in functional differences (Table 3, Figure 2).

We distinguish between isoform-specific differences which impact STIM1/Orai coupling, Orai activation or both (Table 3, Figure 2).

### 4.1. Isoform-Specific Differences That Determine Direct STIM1/Orai Coupling

The major STIM1 coupling site, the Orai C-terminus, contains supercoil regions that differ among the three Orai homologues in their affinity to STIM1, as we demonstrated shortly after the identification of the STIM and Orai proteins via site-directed mutagenesis [98]. This difference underlies their distinct leucine patterns in the sequence of their C-termini [98] (Figure 2A–D). A single point mutation in the Orai1 C-terminus (L273S/D) or the counterpart in STIM1 C-terminus (L373S) was sufficient to abolish the STIM1/Orai1 interaction. In contrast, the interaction of STIM1 with Orai2 or Orai3 and their activation could only be completely inhibited by double point mutations, either in the Orai or the STIM1 C-termini [43,93,98,99] (Table 3 and Table 4). We discovered that the deletion of _277_AEF_279_ (Table 3 and Table 4) in Orai1 C-terminus changes the leucine pattern, which is, thus, comparable to that of Orai2 and Orai3 (Figure 2D). We linked these observations using bioinformatic prediction methods to an enhanced probability for coiled-coil formation in Orai2, Orai3 and the Orai1 Δ_277_AEF_279_ compared to that of Orai1 [98]. Our findings were recently confirmed by peptides from the C-termini of the three distinct Orai proteins attached to a Lyn kinase region anchored to the cytosolic side of the plasma membrane by fatty acylation (LK-CFP-O1/2/3-M4x). Indeed, SOAR dimers colocalized much more strongly with the Lyn kinase peptides containing the C-termini of Orai2 and Orai3 than those of Orai1. Furthermore, STIM1 proteins showed strong interaction with the Orai3 peptide as well as the Orai3 channel, which was completely inhibited only by a double point mutation of the leucine residues (L282D/L285D). In competition experiments, only the LK-O3-M4x, but not the LK-O1-M4x peptide, could block store-operated Ca^2+^ entry in HEK 293 cells. This difference in binding affinity to STIM1 C-terminus may be critical in pharmacological attempts of interference [99].

In addition, we identified an isoform-specific behavior of the loop2 with respect to its communication with the N-terminus [60] as well as in connection with TM3 [103], which will be discussed in the Section 4.3. This likely underlies the isoform-specific differences in the loop2 regions (e.g., Orai1 K161 and analog Orai3 H136, Orai1 E166 and analog Orai3 Q141) (Figure 2A–D). Moreover, we reported that the loop2 segment (aa 160–170) is a critical STIM1-sensitive gating site for Orai1 [97]. For Orai1, it is known that STIM1 L402D/C mutants still allowed coupling with but abolished gating of Orai1. Remarkably, cysteine crosslinking of STIM1 L402C and Orai1 E166C by diamide allowed activation (Table 3), which could be reversed by a break of disulfide bonds via BMS. However, it is currently unknown to which extent these isoform-specific differences affect the interaction with STIM1 and Orai gating. For instance, the question remains whether the anologue residue, though non-conserved (Q141), in Orai3 allows the same effects.

### 4.2. Isoform-Specific Differences in Both STIM1/Orai Interplay and Orai Activation

The C-terminus of Orai1 is connected to TM4 via a bent region called nexus [82,83]. This region has been reported to be critical for STIM1 coupling as well as pore opening. Mutations within this region can lead to constitutive activity and interference with STIM1 coupling [82,83]. This suggests that these mutations induce a conformational change in both the entire channel complex and the C-terminal coupling site. The region is not fully conserved among the three Orai proteins (Orai1: _261_LVSHK_265_, Orai2: _222_LVRHK_226_, Orai3: _270_LVAHK_274_) (Figure 2A–D, Table 3). It is reasonable to assume that the serine, arginine or alanine in the center of the nexus could lead to different functional effects, but further studies are needed in this regard.

We reported that an opening-permissive communication of the N-terminus and loop2 is essential for STIM1/Orai1 interaction and Orai1 activation [94]. Using a series of N-terminal deletion mutants, we uncovered that the interplay of the N-terminus and the loop2 is defined in an isoform-specific manner. Although the Extended Transmembrane Orai1 N-terminal region (ETON) region is identical among the Orai homologues, we discovered that Orai3 necessitates a 5 amino acid shorter portion of the N-terminus than Orai1 to maintain STIM1-mediated activation [94] (Figure 2D). This is caused by the different properties of the Orai loop2 segments. We demonstrated, using Orai1/Orai3 chimeras, that STIM1 interaction and activation of the inactive Orai1 N-truncation mutants, for instance Orai1 ΔN_1–78_, was restored upon the exchange of the loop2 by that of Orai3 (Orai1 ΔN_1–78_ Orai3-L2) [60]. Similarly, the replacement of five non-conserved amino acids within the loop2 of Orai1 with that of Orai3 restored the current activation of the N-truncation mutants by STIM1. Consistent with these results, the function of analogous active Orai3 N-truncation mutant (e.g., Orai3 ΔN_1–53_) was abolished by inserting Orai1 loop2 in place of that of Orai3. In line with these studies, constitutive Orai1 mutants that lost their activity by truncation of the N-terminus also showed restored activity after the swap of Orai3 loop2, independent of STIM1 [60]. Using MD simulations, we demonstrated that the isoform-specific functional differences underlie different structural features of the loop2 regions. Orai1 is predicted to contain a longer flexible loop2 portion, which consequently leads to a shorter cytosolic helical extension of TM2 (Table 3). In contrast, Orai3 possesses a shorter flexible loop2 segment, whereas the TM2 helix extends further into the cytosol (Table 3). Altogether, these distinct structural features define the interplay of the N-terminus with loop2.

We recently showed that inter- and intra-subunit salt-bridge interactions at the cytosolic helical extensions of TM1, TM2 and TM3 are critical for STIM1 coupling as well as pore opening (Figure 2A,E). A single defect of one of the charged residues in the so-called cytosolic triangles blocked pore opening, not only in the presence of STIM1, but also of different GoF mutations already in the absence of STIM1. We showed that these LoF mutations in the cytosolic triangles interfere not only with STIM1 coupling, but predominantly with an opening-permissive pore hydration [81]. These residues which establish the salt-bridge interactions (Table 3) likely regulate the interplay of the N-terminus and the loop2. However, as they are fully conserved among the Orai isoforms, it remains unclear to which extent their interplay might be affected by isoform-specific structural differences of the loop2, as identified in MD simulations [60].

The nexus region is in close proximity to the loop2-TM3 region, forming the hinge plate [82]. Two conserved leucines in TM3 (L174) and the nexus (L261) were reported to fine-tune Orai1 activation [82,83] (Figure 2A–D, Table 3). Whether isoform-specific differences of the loop2 region [60] lead to an altered communication within the hinge plate of Orai2 or Orai3 still requires additional investigations. From these observations, it can be assumed that the loop2 impacts the interplay with the hinge/nexus region in an isoform-specific manner. Indeed, in contrast to a set of other GoF Orai1 mutants, we showed that the constitutive Orai1 hinge mutant (Orai1 ANSGA) loses its activity upon N-terminal deletion, independent of whether the loop2 of Orai3 is swapped or not [104]. The gating checkpoints responsible for this difference are still elusive. Additional investigations are also needed to determine the impact of these isoform-specific differences on STIM1 coupling and STIM1-induced Orai gating.

Moreover, the Orai N-terminus controls CRAC channel activation and biophysical characteristics in an isoform-specific manner, as reviewed in detail in Krizova et al. [92]. Noteworthy, the STIM1-mediated Orai1 currents are 2–3-fold higher compared to those of Orai2 and Orai3 [67,92,98]. These isoform-specific current levels likely underlie polybasic- and proline-rich domains only present in the Orai1 N-terminus [105,106]. Their mutation [54,93,106] significantly lowered Orai1 Ca^2+^ currents to levels comparable to those of Orai2 and Orai3. In support, Orai2 and Orai3 chimeras containing the swapped Orai1 N-terminus showed a marked increase in currents [106].

Aside from the store-operated activation of highly Ca^2+^ selective currents, other common CRAC channel hallmarks include the fast Ca^2+^-dependent inactivation (FCDI) and the increase in I_DVF_ versus I_Ca^2+^_ [92,95]. FCDI is a cellular feedback mechanism to limit the amount of Ca^2+^ influx into the cell. Interestingly, FCDI, which is typically recorded within the first 100 ms of a hyperpolarizing voltage step of 2 s [67,84], occurs in an isoform-specific manner for the three types of Orai channels. The extent of FCDI of STIM1-mediated Orai1 currents is approximately 20%, while that of Orai2 and Orai3 is strongly pronounced, amounting to 50–60% [67,84,107]. Moreover, subsequent to the fast inactivation phase, STIM1-mediated Orai1 currents typically exhibit a reactivation phase which reaches its maximum after ~1500 ms, while that of Orai2 and Orai3 is followed by a slow inactivation phase [67,84,107]. These differences arise due to structural differences within the cytosolic regions of Orai channels [107,108]. Specifically, an exchange of the C-terminus by that of another isoform or the removal of the N-terminal proline/arginine rich region can alter the profile of FCDI [107,108]. Moreover, the N- and C-terminal strands as well as the loop2 region cooperatively regulate FCDI [108]. The extent of FCDI of native CRAC currents is more pronounced than that of STIM1/Orai1 currents and the reactivation phase is lacking. This suggests potential heteromeric Orai channel formation, although the behaviour cannot be fully explained by the 1:1 ratio of Orai1:Orai3 heteromers [109,110]. A potential explanation is that Orai heteromers of subunit composition with different ratios or accessory proteins are further involved in the regulation of FCDI, which still requires additional investigations.

Concerning the typical enhancements of CRAC channel currents when switching from a Ca^2+^-containing solution to a divalent free Na^+^-containing solution, it is of note that the ratio of I_DVF_ versus I_Ca^2+^_ differs among the three isoforms. The reason for that likely underlies the distinct extents of FCDI. Indeed, the pronounced inactivation of STIM1-mediated Orai3 currents corresponds to an increased ratio of I_DVF_ versus I_Ca^2+^_ compared to Orai1 [111].

The isoform-specific properties of the N-terminus are further crucial in the formation of ARC channels, which have been reported to form pentameric assemblies of three Orai1 and two Orai3 subunits. A chimeric approach revealed that the substitution of a single Orai3 N-terminus by that of Orai1 switched the channel into a more store-operated than an arachidonate-activated one [90].

### 4.3. Isoform-Specific Differences in the Mechanisms of Orai Complex activation

The activation signal of STIM1-Orai coupling at the periphery of the channel complex is finally transmitted to the pore region in the center. A series of GoF mutations, each located in one of the four transmembrane domains (TMs), suggested that pore opening involves structural alterations throughout all TM domains [60,71,79,80,81,92,95,104,112,113,114,115,116,117,118,119,120]. Using a library of double mutants, each combining one GoF and one LoF point mutation, we recently showed that the LoF mutation always acted dominantly, thus leading to the abolished activity of the respective double mutants. Hence, we proved that pore opening requires a global conformational change of the entire channel complex [12,81,103]. This involves a series of gating checkpoints to adopt an opening permissive conformation. A single defect in one of these gating checkpoints independent of their location relative to the pore leads to a loss of function and abolished pore dilation [12,81,103]. A comparison of Orai1 and Orai3 revealed that most of the critical gating checkpoints are conserved [103]. We showed that both channel isoforms necessitate an intact allosteric communication of all TM domains to allow pore opening [103].

Despite comparable general gating mechanisms, two non-conserved gating checkpoints in TM3, in particular, V181 and L185 in Orai1 and the corresponding positions A156 and F160 in Orai3, affect pore opening to varying degrees [60,95,103]. Using alanine substitutions, we discovered slight constitutive activity for Orai1 V181A and Orai1 L185A, while Orai3 F160A led to strong constitutive Ca^2+^ entry (Figure 2A–C, Table 5).

Interestingly, the A156 in Orai3 maintains its resting state, despite Orai1 V181A leading to constitutive activity. We identified that the overall hydrophobicity along TM3 determines whether the Orai channels remain in the quiescent state or open already independent of STIM1. Indeed, Orai3 F160L, decreasing the overall hydrophobicity along TM3, led to weak constitutive activity. Moreover, for instance Orai3 A156W F160A, also reducing overall hydrophobicity along TM3, significantly diminished constitutive activity compared to Orai3 F160A [103] (Table 5). Interestingly, while Orai3 F160A led to huge constitutive activity, Orai1 V181A L185A, mimicking two alanines at the two non-conserved checkpoints, still exhibited low constitutive activity; however, both Orai1 and Orai3 contain comparable hydrophobicity along TM3. We identified that in addition to TM3, the loop2 also functions as a critical determinant in manifesting the extent of pore opening. Indeed, constitutive activity of Orai3 F160A was strongly reduced upon the swap of Orai1-loop2, while that of Orai1 V181A L185A was enhanced to levels of Orai3 F160A [103] (Table 5).

Moreover, strongly enhanced hydrophobicity at the analogue positions Orai1 V181 and Orai3 A156 also led to distinct effects. While Orai1 V181F retained plasma membrane expression, but exhibited LoF, Orai3 A156F led to loss of plasma membrane expression [12,81,103] (Table 5).

Interestingly, Orai1 V181K led to robust constitutive activity comparable to Orai3 F160A. These isoform-specific hydrophobic residues are located opposite to hydrophobic amino acids in TM4. At this point, additional investigations are required to clarify how substitutions of amino acids with large, hydrophobic side chains to those with small or to large hydrophilic side chains affect the interplay with TM4 and subsequent pore opening.

The ion conduction path is established by part of the loop1, the so-called CAR, and TM1. Due to full conservation of TM1, the Ca^2+^ ion pore exhibits only isoform-specific differences in the CAR, which consists in Orai1 of three glutamates, while in Orai2 and Orai3, a mixture of glutamates, glutamine and aspartates occurs, as shown in Table 2. This difference does not lead to altered functional properties of homomeric channel complexes. However, heterooligomeric complexes composed of Orai1 and Orai3 display reduced selectivity for Ca^2+^ and increase permeation of Cs^+^ [56,84], indicating a larger diameter of the channel pore.

Basic residues in the loop3 region have been reported to interact with loop1. Their interaction has been shown to compete with Ca^2+^ binding [72] (Table 2). Since neither the residues in the CAR region nor the loop3 is conserved among the three Orai homologues, it might be assumed that these differences lead to Ca^2+^ permeation across Orai channels in an isoform-specific manner.

Overall, the C-termini of the three Orai channels exhibit differences in the coupling to STIM1. Several cytosolic portions, including the N-terminus, loop2 and the nexus region, control STM1 binding as well as Orai pore opening in an isoform-specific manner. Pore opening and Ca^2+^ permeation are regulated by TM3 and the extracellular loop1 and loop3 distinctly among the three homologues.

## 5. Orai Isoform-Specific Sensitivity to pH

Alteration in extra- or intracellular pH regulates most ion channels, which is critical in the development of diseases such as cancer. Extracellular and intracellular acidification blocked, while alkalization promotes, both endogenous as well as overexpressed CRAC channel currents [100,121,122,123]. Sites identified as sensitive to changes in extracellular pH were D110, D112, E106 and E190. H155 located in the intracellular loop has been shown to mediate sensitivity to intracellular pH [123]. All these residues are conserved among Orai channels. Furthermore, alterations in pH have been shown to change activation kinetics, inactivation and voltage-dependence [121,122].

No significant differences in pH sensitivity have been observed for heterologously expressed Orai isoforms upon activation via STIM1 [100]. However, recently, isoform-specific differences in pH sensitivity have been discovered in Orai knock-out (KO) cells, thus containing none of the three Orai isoforms endogenously. Whereas extracellular or intracellular acidification inhibited Orai2 currents similar to those of Orai1, Orai3 currents were unaffected compared with physiological pH. Intracellular and extracellular alkalization enhanced Orai1 and Orai2 currents, while only a marginal effect was observed for Orai3. These studies suggest that pH sensitivity is further influenced by non-conserved sites among Orai isoforms, which remains to be elucidated [101] (Table 3).

## 6. Isoform-Specific Differences in Redox Sensitivity

Reactive oxygen species (ROS) are essentials triggers in a variety of physiological and pathophysiological processes. They are generated outside as well as inside the cell and are produced by redox-active proteins. It has been reported that hydrogen peroxide (H_2_O_2_) blocks STIM1-mediated Orai1, but not Orai3 currents (Figure 2A–C). The reason for that is a non-conserved cysteine (C195) located close to TM3 at the extracellular side. A serine substitution at C195 (Orai1 C195S) reduced redox sensitivity. Orai3 contains at this analogue position glycine (G170), which makes it insensitive to oxidative stress [102]. Substitution of the glycine by a cysteine in Orai3 (Orai3 G170C) makes it redox sensitive [102] (Table 3). Isoform-specific redox sensitivity has been reported to play a critical role in the immune response [124] (as outlined in Section 9). Remarkably, concatemeric studies revealed that a single Orai3 subunit within an Orai1 channel complex is sufficient to transfer insensitivity to oxidative stress [125].

Mechanistically, oxidation of Orai1 has been shown to reduce subunit interaction and slow diffusion. The oxidized cysteine (C195) as well as its oxidomimetic mutation C195D were demonstrated to undergo inhibitory interactions with S239 located in TM4 [125]. Mutation of the serine in Orai1 TM4 abolished H_2_O_2_ mediated inhibition.

## 7. Isoform-Specific Pharmacological Profiles of Orai Channels

A set of compounds modulating Orai channels are currently available. Although none of those has been used in clinics in recent years several ones have reached phases of clinical trials. Most blockers have been reported to possess poor selectivity on Orai channels [17,18].

The most commonly used inhibitors are the trivalent lanthanides La^3+^ and Gd^3+^, which selectively block Ca^2+^ ion channels at low concentrations. They lead to an equal and significant block of different Orai channel isoform currents [10,17,18].

2-APB represents the most popular drug in the characterization of the structure/function relationship of CRAC channels [10,67,126]. Its unique feature is the ability to discriminate between the Orai isoforms due to their distinct pharmacological responses (Figure 2F). Native CRAC currents as well as STIM1-mediated Orai1 and Orai2 currents are inhibited by 2-APB. In contrast, Orai3 exhibits upon application of 2-APB huge double rectifying currents independent of STIM1. The pronounced effects of 2-APB on Orai3 have been attributed to their effect on the pore region. The pore diameter of Orai3 enhances from 3.8 Å to 5.34 Å [66]. A mutation in the TM3 of Orai3 E165Q resembles the permeation properties of 2-APB activated Orai3. The unique action of 2-APB on the pore suggests that this compound interferes with the area around the pore in TM2, TM3 and TM4 [66,127,128,129]. Among the diverse Orai1/Orai3 heteromers, it has been shown that a single Orai3 subunit within an Orai1 complex does alter the 2-APB response compared to wild-type Orai1. In contrast, two or more Orai3 subunits in complex with those of Orai1 led to double rectifying currents, as known for wild-type Orai1 [130].

Two derivatives DPB162-AE and DPB163-AE affected store-operated Ca^2+^ entry in a comparable manner to 2-APB [131,132]. Both inhibited STIM1-mediated Orai1 currents and partially those of Orai2. However, Orai3 currents were not activated by these derivatives as observed with 2-APB, potentially due to their larger size, relative to 2-APB, which potentially impairs their access to the pore [133].

Recently, a library of 2-APB derivatives has been synthesized containing substitutions at one of the phenyl rings of 2-APB. Among those, there are ones which inhibit store-operated Ca^2+^ entry already at low concentrations. As the effects of these novel compounds were investigated on native SOC of breast cancer cells (MDA-MB-231) and HEK 293 cells, their effects on Orai1 and Orai3 individually remain of interest to determine potential isoform-specificity [134].

Despite 2-APB showing an isoform-specific differences in its action on the three Orai homologues, it belongs to one of the most unselective blockers. Several other available blockers, such as Synta66, GSK7975A or RO2959, are rather selective to Orai channels, as they show almost no significant inhibitory effect on a variety of other cellular receptors, transporters or ion channels. Among those, RO2959 blocks STIM1-mediated Orai2 and Orai3 currents to a weaker extent than those of Orai1 [135]. This makes it a very promising candidate for selective interference with Orai1. For Synta66 or GSK7975A, such a pronounced isoform-specific effect has not been observed in overexpression systems, such as HEK 293 cells. However, a recent study using Orai1/2/3-triple KO cells, which possess the advantage of being uncontaminated by endogenous Orai homologues, unraveled isoform-specific properties of CRAC channel inhibitors on individually expressed Orai proteins. They discovered that GSK-7975A and BTP2 inhibit the activity of Orai1 and Orai2, while Orai3 currents were only partially inhibited. Synta66 was identified to abrogate the function of Orai1, while it enhanced the activity of Orai2 and left Orai3 unimpaired. Orai dimers composed of Orai1-Orai1, Orai1-Orai2 and Orai1-Orai3 were blocked by Synta66, while Orai2-Orai3 dimers were not affected. The CRAC channel agonist IA65 exhibited a potentiating effect on Orai1 currents, while those of Orai2 and Orai3 remained unaffected. Recently, molecular docking and live cell studies have identified the Synta66 binding site near the selectivity filter on the extracellular side close to the transmembrane domain (TM)1 and TM3 and the two loop regions. Particularly critical amino acids are in loop1 (H109, H113, Y115) and in loop3 (F199, P201 and L202). Triple point mutations in loop1 (H109D, H113G, Y115G) and loop3 (F199G, P201G, L202G) were predicted to disrupt Synta66 coupling. The mutants showed store-operated activation of nonselective currents in the presence of STIM1, which were only partially inhibited by Synta66. A comparison of these critical amino acids in Orai1 loop1 with the analogous ones in Orai2 and Orai3 revealed that they are almost fully conserved, except for H113, which is a tyrosine in Orai2. In Orai1 loop3, F199 and P201 are fully conserved, while L202 in Orai1 is conserved as hydrophobic amino acid and corresponds to alanine in Orai2 and isoleucine in Orai3 [136]. Hence, the key residues specific for Synta66 binding are largely conserved. It remains to be elucidated whether the small differences in sequence or additional sites are responsible for the recently reported isoform-specific effects of Synta66 [101].

Altogether, isoform-specific differences have been discovered for several available pharmacological compounds (Figure 2F). The characterization of their detailed mode of action and binding pockets would help to optimize available drugs or to generate novel target-specific ones.

## 8. Orai Isoform-Specific Downstream Signaling

Cellular Ca^2+^ signals are prominent for the versatility to regulate a variety of signal processes ranging from short to long term response. This is established by the spatiotemporal dynamics of calcium signaling patterns, including transient, oscillatory or robust ones [2,137,138,139,140].

Orai1 is mainly indispensable for the development of Ca^2+^ plateaus involved in sustained Ca^2+^ entry [85,141,142]. Orai2 and Orai3 were shown to be essential for the generation of Ca^2+^ oscillations triggered by physiological receptor stimulation, whereas they are not involved in sustained Ca^2+^ entry. Indeed, various Orai isoform knockout cells showed that only Orai1-containing cells predominantly exhibited sustained Ca^2+^ entry. In contrast, KO cells with sustained expression of Orai2 or Orai3 either alone or together with other isoforms showed oscillatory Ca^2+^ level increases. Interestingly, a lower oscillation frequency was detected in cells containing only Orai2 or Orai3 alone compared with cells containing two or three isoforms. This isoform-specific behavior correlates with an enhanced STIM1 coupling to Orai2 and Orai3 compared to Orai1 in the resting state as well as with their isoform-specific FCDI, which is the highest for Orai3 [85].

**Figure 3:**
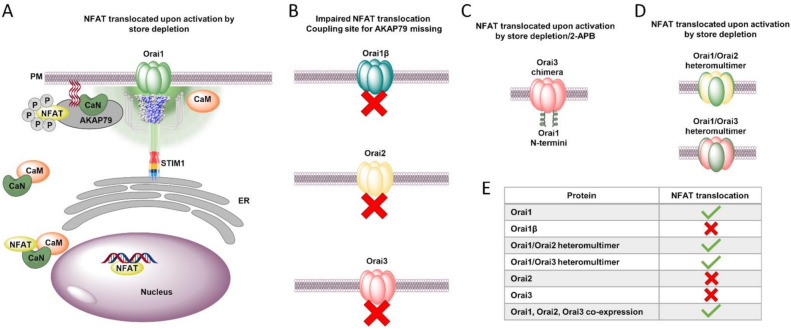
Nuclear factor of activated T-cells (NFAT) translocation pathway in dependence of Orai isoforms. (**A**) NFAT translocation is initiated by Orai1 Ca^2+^ influx but not that of other Orai isoforms. The STIM1-activated Orai1 channel triggers a local Ca^2+^ influx, which is sensed by the Ca^2+^ sensor calmodulin (CaM, orange). Ca^2+^-bound calmodulin enables binding to the adjacent calcineurin (CaN) rapidly released by AKAP79. The cytosolic complex of Ca^2+^/calmodulin-calcineurin recognizes the phosphorylated NFAT and binds to its consensus sequence. In the following, NFAT is dephosphorylated by CaN and subsequently translocated into the nucleus where it binds to its recognition site on the DNA. (**B**) The NFAT translocation is impaired (red cross) for Orai1β, Orai2 and Orai3 due to the missing coupling site for AKAP79. (**C**) Chimeric mutant with Orai1 N-terminus recovers the impaired NFAT translocation of Orai3. (**D**) Orai1/Orai2 and Orai1/Orai3 allow NFAT translocation. (**E**) These requirements for NFAT translocation are summarized in the table (green check mark: NFAT translocation occurs; red cross: NFAT translocation does not occur).

Local elevation in Ca^2+^ levels can be translated into various output signals by diverse effector molecules. Among the latter, we focus in this section on the nuclear factor of activated T-cells (NFAT), typically found in the cytosol in the vicinity of the Ca^2+^ source, and responsible to convey the signal of Ca^2+^ influx to the nucleus to trigger gene transcription. This is essential for triggering the innate and adaptive immune cell response through the transcription of inflammatory mediators such as cytokines. The four members of the NFAT family, NFAT1–4, are kept inactive in the cytosol by phosphorylation. The movement of NFAT into the nucleus is triggered by local Ca^2+^ entry [139,143,144,145,146,147,148,149,150,151]. For the Orai isoforms, Ca^2+^-triggered NFAT movement into the cytosol was shown to be specific for Ca^2+^ influx via Orai1 and absent for Orai2 and Orai3 [152]. Orai1-mediated gene transcription involves calmodulin, a versatile Ca^2+^ binding protein, calcineurin, a Ca^2+^-dependent phosphatase, and AKAP79, a prototypical A-kinase anchor protein. Mechanistically, Ca^2+^ entry leads to the attraction of calcineurin to calmodulin bound to Orai1 via the scaffolding protein AKAP79. AKAP79 binds to NFAT1 via its C-terminal leucine zipper motif, thus bringing it in close proximity to Orai1 upon Ca^2+^ entry (Figure 3A,E). AKAP79 is bound to the N-terminus of Orai1 in the region of aa39–59 [152]. Deletion of this segment abolishes NFAT translocation. Orai2 and Orai3 lack this AKAP79 coupling site due to their much shorter N-terminus compared with Orai1, making them unable to translocate NFAT. Furthermore, Orai1ß, in which the first 63 aa are missing, exhibited impaired NFAT translocation (Figure 3B,E). Remarkably, an Orai3 chimera containing Orai1 N-terminus enabled NFAT translocation (Figure 3C,E), triggered by both store-depletion [152] and 2-APB [147]. A peptide mimicking the AKAP79 binding site of Orai1 was able to suppress activation of NFAT1 by leukotriene receptor and inhibited cytokine production, but left other Orai1-dependent functions, such as secretion, unaffected [152].

These isoform-specific properties in producing Ca^2+^ signals and gene transcription are crucial for extending the bandwidth of Ca^2+^ signals, and thus for fine-tuning downstream signaling events. Moreover, this is enabled by Orai heteromultimers consisting of Orai1 combined with Orai2 and/or Orai3. This was demonstrated using NFAT translocation assays. While activation of Orai1 enables nuclear translocation of NFAT1 and NFAT4, stimulation of Orai2 or Orai3 keeps these transcription factors in the cytosol (Figure 3B,E). Remarkably, cells coexpressing Orai1 with Orai2 and/or Orai3 enabled NFAT translocation (Figure 3D,E). This led to the conclusion that the isoform-specific differences of Orai homologues, together with their diverse heteromeric assemblies, as well as the strength of agonist stimulation, allow the generation of a variety of distinct Ca^2+^ signals capable of specifically triggering diverse cellular downstream signaling processes [85].

In summary, Orai isoforms define the pattern of Ca^2+^ signals which develop in the cells and subsequent downstream signaling pathway. Interestingly, STIM1-mediated Orai3 Ca^2+^ entry is unable to induce NFAT triggered gene transcription.

## 9. Physiology and Pathophysiology of Orai Isoforms

Orai1, suggested to be part of the “classical” CRAC channel, exhibits a pervasive expression in many cell types including immune cells, cardiomyocytes, vascular smooth muscle cells, endothelial cells, melanocytes and airways [62,153,154]. In particular, Orai1 controls a broad variety of immune system functions [155,156,157]. The most prominent example for the essential role of Orai1 in the immune system is exemplified by a single point mutant (Orai1 R91W), leading to severe combined immune deficiency [19] (Table 6).

**Figure 4:**
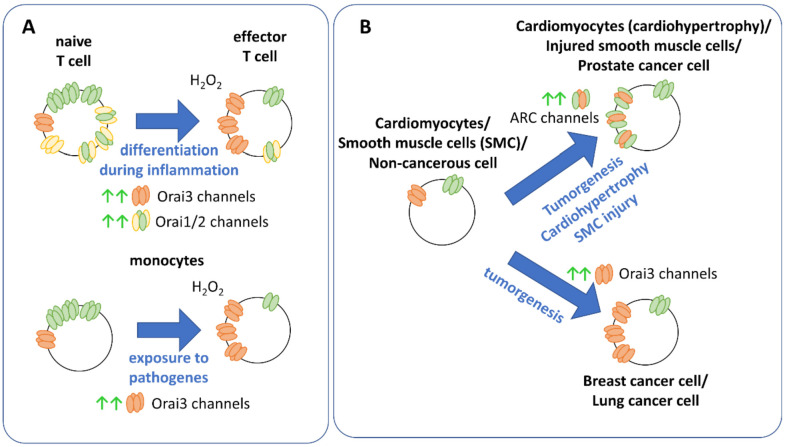
Pathophysiological relevance of Orai2 and Orai3 channels in T-cells and cancer. (**A**) Enhanced Orai3 expression occurs upon inflammation in T cells and upon exposure to pathogens in monocytes. Furthermore, Orai2 levels are enhanced in T cells upon inflammation. (**B**) While prostate cancer cell development, smooth muscle cell injury or cardiohypertrophy is associated with an increase in Orai3-Orai1 constituted heteromultimeric ARC/LTC4 channels, breast/lung cancer cell development is linked to a significant increase in Orai3 expression. (yellow: Orai1, green: Orai2, orange: Orai3).

Orai2 and Orai3 and their isoform-specific differences broaden their roles in the healthy human body and in disease. They are critical to fine-tune immune cell response, cardiac and muscle function. Orai2 is mainly expressed in the brain and at lower levels in the spleen, lung and small intestine. Orai3 occurs similar to Orai1 in the brain, heart, lung, kidney, skeletal muscle and other organs [19,62,181]. Moreover, especially Orai3 has been identified to initiate cancer cell development [160,161]. An upregulation of Orai2 or Orai3 versus Orai1 typically offers a survival advantage of a certain cell type under specific conditions, as outlined below [85] (Figure 4, Table 6).

In regard to immune cell response, different expression levels of Orai1 and Orai3 have been reported to tune the cellular response of T cells under oxidative stress. Typically, Ca^2+^ entry into T lymphocytes controls their activation, proliferation and differentiation. Inflammatory processes trigger the migration of T helper (T_H_) cells to the respective sites and induce their differentiation into effector T_H_ cells. The latter proliferate and secrete cytokines. It has been demonstrated that CRAC channels in naïve T helper cells are more susceptible toward H_2_O_2_ than those of effector T helper (T_H_) lymphocytes. The reason for this is that Orai3, which is not redox sensitive, is upregulated in the effector T_H_ cells (Figure 4A). Thus, these cells developed an adaptive mechanism to be able to continue proliferation and cytokine production when levels of ROS are high, such as during inflammation. Otherwise, in the absence of Orai3, high levels of ROS would inhibit T cell activation [102,161]. A similar switch to enhanced Orai3 expression has been reported for monocytes (Figure 4A), which are effector cells of the innate immune system and involved in the elimination of pathogens and the stimulation of other immune cells, when exposed to pathogens. They are typically more suspended to oxidative stress than T-cells. It has been shown that redox stress enhances the expression of Orai3, to make monocytes resistant to redox-triggered inhibition. Indeed, knockdown of Orai3 increases the sensitivity of monocytes to redox stress [124] (Table 6).

Additionally, Orai2 has also been reported to fine-tune store-operated Ca^2+^ entry in T cells. While it is highly expressed in naïve T cells, it is downregulated in effector T cells. The enhanced ratio of Orai1:Orai2 in naïve T cells (Figure 4A) reduces store-operated Ca^2+^ entry likely due to the pronounced inactivation of Orai2 compared to Orai1. Indeed, knockdown of Orai2 enhanced, while deletion of Orai1 reduced store-operated Ca^2+^ currents. Only the deletion of both Orai1 and Orai2 blocked store-operated Ca^2+^ entry and drastically interfered with T cell function [56] (Table 6).

Cardiomyocytes are essential to trigger a contractile force in the heart. Cardiac hypertrophy has been associated with an enhanced interplay of Orai1 and Orai3 (Figure 4B), in contrast to healthy cardiomyocytes, although ARC channels are also expressed there [162,163]. Loss of Orai3 expression has been reported to lead to ventricular dysfunction progressing to dilated cardiomyopathy and heart failure. Orai3 is required for proper heart function, maintains intact sarcomere formation and mitochondrial function [164] (Table 6).

Smooth muscle cells control the function of a series of hollow organ systems, such as vasculature, airways or uterus [182]. STIM and Orai proteins have been reported to constitute molecular key components mediating store-operated Ca^2+^ entry in these cells. However, an altered expression level or function of Orai channels has been connected to diseases such as hypertension or atopic asthma [165,166]. In the resting state, vascular smooth muscle cells do not proliferate or migrate. However, upon mechanical injury, they can switch to highly proliferative and migratory cells. Under these conditions, VSMCs exhibit increased Orai3 expression levels, where it does not contribute to store-operated Ca^2+^ entry, but to a store-independent Ca^2+^ entry pathway (Figure 4B, Table 6) activated by thrombin. The latter involves ARC channels [26,167]. Furthermore, ARC channels have been reported to play a role in airway smooth muscle cells. Patients suffering from asthma exhibited enhanced expression of this channel type compared to corresponding healthy cells [165,168].

The activity of astrocytes, which represent non-excitable cells in the brain, largely depends on cytosolic Ca^2+^ concentrations. Both Orai1 and Orai3 are expressed in astrocytes and together with STIM1, they contribute to robust store-operated Ca^2+^ entry (Table 6). However, their role in physiology as well as in pathophysiology, for instance, the development of Alzheimer, where astrocytes exhibit drastically enhanced Ca^2+^ entry, still requires intense investigations [169].

An increasing number of reports demonstrates a unique and predominant role of Orai3 in cancer development among all three Orai channel family members. Besides the homomeric Orai3 channel, additionally, ARC or LTC4 channels, both heteromeric assemblies of Orai1 and Orai3, have been reported to trigger the development of certain types of cancer cells (Table 6).

Especially for estrogen receptor-expressing (ER+) breast cancer and non-small cell lung cancer (NSCLC), there is ample evidence that Orai3 functions as an attractive therapeutic target in these pathogenic cell types. In contrast to healthy breast tissue, estrogen receptor positive (ER+) breast tumorigenesis exhibit store-operated Ca^2+^ entry encoded by Orai3 [27,170] (Figure 4B). Indeed, Orai3 overexpression in these cells leads to its activation by STIM proteins, while downregulation of Orai3 blocked native store-operated Ca^2+^ and CRAC channels [27,161,171,172]. Moreover, Orai3 has been reported to be essential in the regulation of the cell cycle, as knockdown of Orai3 leads to cell cycle arrest in G1 phase [173]. Furthermore, Orai3 is essential in the proliferation and migration of estrogen receptor-expressing cells [170,171,173]. Orai3 expression is regulated by the estrogen receptor- (ERα), as its downregulation reduced Orai3 levels as well as store-operated Ca^2+^ entry. The latter were rescued by Orai3 overexpression. The critical role of Orai3 in cell survival of breast cancer cells came also clear upon siRNA delivery via nanoparticles, which decreased the viability of breast cancer cells (T47D) [174], but not of healthy cell types (MCF10A). Moreover, transient receptor potential canonical 6 (TRPC6) channel has been reported to regulate plasma membrane translocation of Orai3 as well as Orai1 in breast cancer cell line [175]. Overall, these studies highlight the significant role of Orai3 to potentially function as a therapeutic target in breast cancers.

Concerning lung cancer cells, in lung adenocarcinoma cells, a higher Orai3 expression (Figure 4B) has been reported compared to noncancerous tissue, both in cell line [28,176] and in native cell samples of 200 patients. It has been reported that the progressive development of cancer cells is connected to an increase in Orai3 expression. Application of 2-APB and knockdown of Orai3 revealed that Orai3 contributes to store-operated Ca^2+^ entry. Furthermore, Orai3 was identified to trigger the proliferation of lung adenocarcinoma cells. Similar to breast cancer cells, in lung cancer cells Orai3 expression has also been demonstrated to be regulated by ERα.

In prostate cancer cells, the expression profile of Orai3 is still controversial. While Dubois et al. [32] demonstrated enhanced Orai3 expression levels, Holzmann et al. [177] suggested a decrease in Orai3 expression. Nevertheless, in both studies, the formation of Orai1-Orai3 heterooligomeric ARC channels has been found to play a role in prostate cancer cell development (Figure 4B). In accordance with this finding, Dubois et al. [32] showed that arachidonic acid triggers the proliferation of prostate cancer cells. Moreover, overexpression of Orai3 enhanced the number of ARC channels together with proliferation, while apoptosis of prostate cancer cells was decreased. Downregulation of Orai1 decreased prostate cancer cell growth, suggesting that both isoforms Orai1 and Orai3 are indispensable to drive prostate cancer cell development. Specifically, they demonstrated that Orai1, but not Orai3, contributes to their store-operated Ca^2+^ entry, though Orai3 is also expressed [32]. Holzmann et al. [177] identified that prostate cancer cells are more sensitive to ROS, which is likely due to Orai1 expression. Although components of arachidonic acid-regulated ion channels are expressed in breast cancer cells, in contrast to prostate cancer cells, arachidonic acid has been associated with more antitumor activity there [178]. Furthermore, there is evidence that Orai3 channels play a role in neuroendocrine tumors, colorectal cancer and leukemia [25,161].

Overall, Orai1 plays an important role in maintaining physiological processes in the human body as well as the individual cell. In contrast, Orai3 in particular is a critical determinant in cells to adapt to altered pathological conditions such as increased oxidative stress or tumorigenesis.

## 10. Conclusions and Perspectives

Collectively, in this review, we have assembled the current knowledge on the structural and functional differences of the three Orai isoforms. Since Orai1 is the most universal ion channel and a variety of disease-related mutations are known, it has been the best characterized to date. However, there is increasing evidence that Orai2 and Orai3 are also of considerable importance, particularly in the development of disease. The universality of Orai1 makes therapeutic approaches difficult to dangerous, as this, in turn, could lead to immunosuppression and promote the development of diseases such as cancer. Hence, understanding the differences of Orai isoforms, both mechanistically as well as physiologically, is critical and may open new avenues for the development of potential future therapeutic strategies that selectively target one of the three homologues.

While the general mechanism of STIM1-mediated activation of the three Orai isoforms is comparable, they can be distinguished in their structural, functional and pharmacological properties. A number of differences in the structure/function relationship of Orai1 in particular compared to Orai3 have been elucidated, such as that of the cytosolic regions. Moreover, Orai1 and Orai3 differ in pH and redox sensitivity. There is evidence of isoform-specific pharmacological modulation of Orai channels. In addition, patterns of Ca^2+^ signaling as well as downstream signaling events, such as altered gene transcription, are differentially regulated by the three isoforms. The range of cellular Ca^2+^ signaling is massively expanded by the possibility of Orai isoform to heteromerize. Although Orai1 is a versatile ion channel in health and disease, there is increasing evidence that cells use a molecular switch mechanism that leads to the up- or downregulation of a particular Orai isoform to adapt to changing physiological or pathophysiological conditions. However, a set of questions still remains to be resolved.

Several functional differences have been extensively characterized, particularly for Orai1 and Orai3. Less attention was paid to Orai2, which mimics some properties of Orai3 (e.g., N- and C-terminus) as well as some properties of Orai1 (e.g., loop3). While the isoform-specific roles of cytosolic segments are well understood, investigations of the role of the second extracellular loop (loop3), which largely differs in sequence identity among the three isoforms, is still pending. Additional efforts need to be made to understand the homo- and hetermomerization mechanisms of Orai subunits and key sites required for that.

With respect to the structural resolutions, only those of dOrai channels in both the closed and open states are currently available. However, for an atomistic characterization of the three human Orai isoforms, their structural resolutions are still outstanding. This could further improve our understanding of the bulk of functional isoform-specific behaviors. The ultimate goal is to obtain structural resolutions of these homologues in complex with STIM1. This would also provide a structural understanding of STIM1/Orai interaction interfaces, obviously occurring in an isoform-specific manner at least for Orai C-termini [98,99]. Herewith, new docking sites for potential drugs could be resolved. Furthermore, structural resolutions of heteromeric assemblies as well as the pentameric ARC channel are of interest. The initial indications of isoform-specific effects of blockers are promising, but still require a solid foundation at the structural level. An atomic level understanding of the binding pockets of available drugs and their isoform-specific differences is still pending, but could help to optimize drugs for site-specificity.

A number of accessory proteins have been identified in recent years to regulate the function of Orai1 channels. However, nothing is known for Orai2 and Orai3 in this regard. The identification of isoform-specific regulatory proteins may open further targets for selective therapeutic intervention, as may be possible for AKAP79 in gene transcription. For example, it is interesting to note that in breast cancer cells, Orai1 has been reported to be regulated by Secretory pathway Ca^2+^-ATPase (SPCA2). Furthermore, however, Orai3 also plays a role in breast cancer cells and is expressed in excess there. It would need to be clarified whether SPCA2 can also regulate Orai3 or whether this is isoform-specific. Another modulatory protein, CRACR2A, has been reported to directly interact with STIM1 and Orai1 to impact their function [183], while no such regulatory effect has been observed for Orai3 [96].

Finally, a detailed understanding of the signaling cascades altering the expression of a certain Orai homologue in disease, as well-known for Orai3 in cancer, is still outstanding. For instance, in breast cancer cells, it has been shown that Orai3 upregulation is controlled by the ERα receptor. Such links might offer valuable targets to interfere with disease states. Furthermore, since NFAT1 mediated gene transcription has been recently reported to be impaired upon store-operated Ca^2+^ entry via Orai3, it remains to be determined how cytokine production in T-cells is possible, potentially via transcription factors, which can be specifically triggered by Orai3.

In terms of therapeutic approaches, it remains crucial to develop not only selective strategies, but also methods of local drug delivery so that potential side effects can be circumvented. Recent advances in the field of nanotechnology have made it possible to deliver chemotherapeutic agents directly to the tumor site. This has significantly reduced the side effects and systemic toxicities related to these drugs [184,185,186]. The use of monoclonal antibodies (mAbs) represents another interesting technology. Excitingly, a mAb was developed that selectively targeted loop3 of Orai1 and abrogated the activity of Orai1 while failing to recognize Orai2 or Orai3 [187,188]. A promising approach would be to target the non-conserved loop3 of Orai3 [25,29]. According to the recent reports, solid evidence that light enables to obtain high spatiotemporal control over the protein of interest is highly emerging. Remarkably, several publications [49,189,190,191] demonstrated the successful transfer of light sensitivity to STIM proteins and lately also to Orai1 using naturally occurring photo-responsive homo-/heteromerization domains. Although it is still technically challenging to precisely control light-sensitive proteins in native tissue, if successful, it would offer an encouraging therapy option. For instance, the use of optogenetic tools that are sensitive to near-infrared light may be promising because they can penetrate deeper into tissue [190,192]. Conclusively, optogenetic strategies could offer the ability to selectively target Orai isoforms.

## Figures and Tables

**Table 1:** Orai domains and their sequence identity among the three Orai isoforms.

Extent of Sequence Similarities of Orai Isoforms		
Structure	Sequence Alignment Orai1 versus Orai2/Orai3 (%)	References
TM1	100	[34]
TM2-TM4	81–87	[34]
NT	34	[29]
CT	46	[29]
loop1	60–80	[52]
loop2	75–87.5	[52,60]
loop3	20–30	[29]

TM … transmembrane domain, NT … N/NH_2_-terminus, C/COOH-terminus.

**Table 2:** Isoform-specific alterations of regions determining Ca^2+^ permeation through the Orai pore.

Residues Determining Ca^2+^ Permeation of Orai Channels					
	Orai1	Orai2	Orai3	function	References
CAR	D110/D112/D114	E84/Q86/Q88	E85/D87/E89	Ca^2+^ attraction	[52,72]
selectivity filter	E106	E81	E82	Ca^2+^ attraction/selectivity	[52]
hydrophobic cavity	L95/F99/V102	L69/F73/V76	L70/F74/V77	rotates to allow pore opening	[52,77]
basic region	G98/R91/K87/R83	G72/R65/K61/R57	G73/R66/K62/R58	maintain closed state or allow Ca^2+^ permeation	[52,61,68,78]
loop1-loop3	D112-R210 cysteine crosslining reduces Ca^2+^ permability	n.d.	n.d.	Modulate Ca^2+^ permeability	[52,72]

n.d. … not determined.

**Table 3:** Isoform-specific differences in regions determining STIM1-Orai1 coupling, Orai1 activation, pH sensitivity and redox sensitivity.

Isoform-Specific Differences in Orai Channel Function				
	Orai1	Orai2	Orai3	
**Coupling to STIM1**				
CT	L273	L237	L285	[43,93,98,99]
	L276	L237 and L244	L285 and L292	
	_277_AEF_279_		L282 and L285	
STIM1 L402	Orai1 E166	n.d.	n.d.	[97]
**Coupling to STIM1 and Orai1 Activation**				
Hinge	_261_LVSHK_265_	_222_LVRHK_226_	_270_LVAHK_274_	[82,83]
Loop2	shorter TM2 extended to cytosol	n.d.	longer TM2 extended to cytosol	[60]
	longer flexible loop2		shorter flexible loop2	
	_151_VSNVHNLNSVKE_162_		_133_NSVHQS_138_	
	accessible for Orai1 ΔN_1–76/78_ (leads to LoF)		inaccessible for Orai3 ΔN_1–51/53_ (retains function	
N-terminus-loop2 communication	K78-E166	n.d.	n.d.	
salt bridge formation (cytosolic triangles)	K85-E173	K59-E147	K60-E148	[81]
	R83-E149	R57-E123	R58-E124	
	K85-E149	K59-E123	K60-E124	
hinge plate	L174-L261	L148-L222	L149-L270	[82,83]
**Sensitivity pH**				
	D110/ D112/ E106/ E190			[100]
acidification	currents inhibited	currents inhibited	currents unaffected	[101]
alkalization	enhanced currents	enhanced currents	currents unaffected	
**Redox Sensitivity**				
H_2_O_2_	blocked currents		currents unaffected	[102]
	C195		G170	

**Table 4:** Isoform-specific differences in the coupling of Orai C-termini to STIM1.

		STIM1 wt	STIM1 L373S	STIM1 L373S A376S
**Orai1**	**wt**	++	-	-
	**Δ_277_AEF_279_**	++	+	-
	**L273D/S**	-	-	-
**Orai2**	**wt**	++	+	-
	**L237S**	+	+	-
	**L237S and L244S**	-	-	-
**Orai3**	**wt**	++	+	-
	**L285S**	+	+	-
	**L285S and L292S**	-	-	-

++ robust store-operated STIM1/Orai activation, + reduced store-operated STIM1/Orai1 activation, no STIM1/Orai1 activation.

**Table 5:** Gain-of-function and loss-of-function mutations and isoform-specific differences of gating checkpoints in Orai TM3.

Gain-of-Function and Loss-of-Function Mutations in Orai TM3					
	LoF	Slight GoF	Robust GoF	Non-Conserved GoF Mutations in TM3	
**Orai1**				**Orai1-TM3**	**Orai3-TM3**
V181A		x		L188	L163
V181F	x			F187	F162
V181K			x	L185	F160
V181A L185A		x		V181	A156
L185A		x		F178	F153
**Orai3**					
F160A			x		
F160L		x			
Orai3 A156W F160A		x			
A156F-loss of PM					
**Chimeras**					
Orai1 Orai3 loop2 V181A L185A			x		
Orai3 Orai1 loop2 F160A		x			

**Table 6:** The expression and pathophysiological relevance of Orai isoforms.

Orai Isoform-Specific Expression and Roles in Physiology and Pathophysiology			
	**Orai1**	**Orai2**	**Orai3**
**Healthy tissue expression**	immune cells, cardiomyocytes, vascular smooth muscle cells, endothelial cells, melanocytes, airways [62,153,154]	brain, spleen, lung, small intestine [11,20,62,158,159]	brain, heart, lung, kidney, skeletal muscle [160,161]
**Immune System** [56,102,124,155,156,157,161]			
**naïve T_H_ cells**	essential	highly expressed	
**effector T_H_ lymphocytes**	essential	downregulated	upregulated
**monocytes**	essential		upregulated
*oxidative stress response*	*redox sensitive*	*n.d.*	*not redox sensitive*
**Cardiac System** [26,162,163,164,165,166,167,168]			
**cardiomyopathy**	essential		loss of expression
**Smooth muscle cells**	essential	n.d.	upregulated
**Brain** [169]			
**astrocytes**	esential		essential
**Cancer** [25,27,28,32,161,170,171,172,173,174,175,176,177,178]			
**Cancer tissue expression**	Renal carcinoma, breast, Melanoma, Glioma, Esophageal squamous cell carcinoma, Pancreatic adenocarcinoma, Prostate	Parathyroid tumors, Prostate	Breast, Prostate, Renal carcinoma, Lung adenocarcinoma
**Disease Related Muations** [15,19,71,80,115,116,117,118,179,180]			
**immunodeficiency (LoF**)	R91W, G98R, A103E, V181SfsX8, L194P	n.d.	n.d.
**Stormorken or Stormorken-like syndrome (GoF**)	S97C, P245L		
**Tubular Aggregate Myopathy (GoF**)	G98S, V107M, L138F, T184M		
**Cancer related (GoF and LoF**)	A137V, M139V, S159L, G183D, G247S		

## Data Availability

No new data were created or analyzed in this study. Data sharing is not applicable to this article.

## Abbreviations

2-APB	2-aminoethoxydiphenyl borate
Å	angstrom (unit of length equal to 10^−10^ m)
aa	amino acid
AKAP79	A-kinase anchor protein
ANSGA	four-point mutation in hinge region aa position 261–265
ARC	arachidonate regulated Ca^2+^ channel
BMS	bis(2-mercaptoethyl)sulfone
BTP2	[N-4-[3,5-bis(Trifluoromethyl)-1H-pyrazol-1-yl]phenyl-4-methyl-1,2,3-thiadiazole-5-carboxamide]
Ca^2+^	calcium ion
CAD	Ca^2+^ release-activated Ca^2+^-activating domain
CAR	Ca^2+^-accumulating region
CC	coiled-coil
CRAC	Ca^2+^ release-activated Ca^2+^
cryo-EM	cryogenic electron microscopy
Cs^+^	cesium ion
ΔN	represents N terminal deletion mutants
dOrai	Drosophila melanogaster Orai
DVF	divalent-free
ER	endoplasmic reticulum
ETON	extended transmembrane Orai1 N-terminal
FCDI	fast calcium-dependent inactivation
FRET	fluorescence resonance energy transfer
GoF	gain of function
GSK	GlaxoSmithKline compounds
I_Ca^2+^_	CRAC current
I_Na+_	sodium current in sodium divalent-free solution
IP_3_	inositol triphosphate
KO	Knock out
LoF	loss of function
LTC4	Leukotriene C4 -regulated Ca^2+^ (LRC) channels
LV(SHK)	hinge region aa position 261–265
MD	molecular dynamics simulations
Na^+^-DVF	sodium divalent free
NFAT	nuclear factor of activated T-cells
NSCLC	non-small cell lung cancer
Orai 1–3	Orai proteins (also O1–3)
PIP_2_	phosphatidylinositol 4,5-bisphosphate
PM	plasma membrane
ROS	reactive oxygen species
SAM	sterile α-motif
siRNA	Small interfering RNA
SOAR	STIM–Orai-activating region
SPCA2	Secretory pathway Ca^2+^-ATPase
STIM	stromal interaction molecule
Synta66	4-Pyridinecarboxamide
TM	transmembrane helices
TRP	transient receptor potential ion channel (C-canonical, M-melastatin, V-vallinoid)
